# Incidence and predictors of COVID-19 and flares in patients with rare autoimmune diseases: a systematic survey and serological study at a national reference center in France

**DOI:** 10.1186/s13075-021-02565-0

**Published:** 2021-07-13

**Authors:** Renaud Felten, Marc Scherlinger, Aurélien Guffroy, Vincent Poindron, Alain Meyer, Margherita Giannini, Anne-Sophie Korganow, Christelle Sordet, Emmanuel Chatelus, Rose-Marie Javier, Aurore Meyer, Luc Pijnenburg, Jean-François Kleinmann, Jacques-Eric Gottenberg, Jean Sibilia, Thierry Martin, Laurent Arnaud

**Affiliations:** 1 Service de rhumatologie, Service de rhumatologie, Hôpitaux Universitaires de Strasbourg et Université de Strasbourg, 1 avenue Molière BP 83049, 67098 Strasbourg cedex, France; 2 Centre National de Référence des Maladies Auto-Immunes Systémiques Rares Est Sud-Ouest (RESO), Strasbourg, France; 3 Service d’immunologie clinique et médecine interne, Hôpitaux Universitaires de Strasbourg et Université de Strasbourg, Strasbourg, France; 4 Service de physiologie et explorations fonctionnelles, Hôpitaux Universitaires de Strasbourg et Université de Strasbourg, Strasbourg, France

**Keywords:** Autoimmune diseases, Epidemiology, Immune system diseases, COVID-19

## Abstract

**Background:**

The risk of severe COVID-19 and its determinants remain largely unknown in patients with autoimmune and inflammatory rheumatic diseases. The objective of this study was to assess the prevalence of COVID-19 infection in patients followed for rare autoimmune diseases as well as the predictors of COVID-19 and disease flare-ups.

**Methods:**

Cross-sectional phone survey from April 9, 2020, to July 2, 2020, during which patients with autoimmune diseases followed at the National Reference Center for Rare Autoimmune diseases of Strasbourg were systematically contacted by phone and sent a prescription for a SARS-CoV-2 serology.

**Results:**

One thousand two hundred thirty-two patients were contacted. One thousand fifty-five patients with a confirmed diagnosis of systemic autoimmune disease were included (4 unreachable, 4 moves abroad, 5 deaths before pandemic, 50 without consent, and 114 without autoimmune disease). Among them, 469 (44.5%) patients were tested for SARS-CoV-2 serology.

Thirty-nine patients (7.9%) had SARS-CoV-2 infection (either through chest CT-scan [n = 5], RT-PCR on nasopharyngeal swab [n = 14], or serology [n = 31]) among the 496 who underwent at least one of those 3 diagnosis modalities. Of the 39 proven cases, 33 had clinical manifestations (6 asymptomatic patients were diagnosed through systematic serology testing), 31 were managed by home care, 3 were hospitalized due to a need for oxygenation, two required admission to an intensive care unit, and one died. Among patients with confirmed SARS-CoV-2 infection, reported flares were more frequent than in uninfected patients (26.3% [10/38] vs. 7.0% [32/457], p < 0.0001). Preventive sick leave had no significant impact on the prevalence of SARS-CoV-2 infection (5.8% [3/53]) compared to work continuation (7.6% [30/397], p = 0.64).

Overall, the seroprevalence of SARS-CoV-2 was 6.6% (31/469) which was numerically lower to the Grand-Est general population estimated to be 9.0%.

**Conclusions:**

This systematic survey of more than 1000 patients with rare systemic autoimmune diseases reports a low prevalence of proven SARS-CoV-2 infection and very rare severe infections, probably related to good compliance with prophylactic measures in these patients.

**Supplementary Information:**

The online version contains supplementary material available at 10.1186/s13075-021-02565-0.

## Introduction

The severe acute respiratory syndrome coronavirus-2 (SARS-CoV-2) pandemic began in December 2019 in Wuhan, China, and then spread around the globe, affecting every continent. At an early stage of the pandemic, chronically ill and/or immunocompromised patients were identified as at-risk patients for the coronavirus disease-2019 (COVID-19). Patients with rare autoimmune diseases, often treated with immunosuppressive drugs, were included in this at-risk group.

The French region “Grand-Est” represents more than 5.5 million people. This area was the most early and the most heavily affected area in France [1]. A wave of contamination emerged at the end of February 2020, leading to a national lockdown between March 17th and May 11th, 2020. Our tertiary center in Strasbourg is the only French national referral center for rare systemic and autoimmune diseases in the “Grand Est” area.

The SARS-CoV-2 is a new virus and COVID-19 a new disease. People with rare autoimmune diseases appropriately confronted their rheumatologists with questions such as their potential increased risk of severe disease and whether lowering immunosuppressive drugs, shielding, and/or preventive sick leave would be appropriate. In this regard, national and international recommendations have been published [2]. However, it is important to rapidly accrue scientific knowledge, and methodologically robust information is crucially needed since COVID-19 is nowhere near extinguished [2].

The main objective of our study was to assess the prevalence of documented and undocumented SARS-CoV-2 infection in patients followed for rare autoimmune diseases and to assess the predictors of COVID-19 as well as the risk of disease flare-ups in the context of potential changes in treatments in these patients. Our secondary objective was to compare this prevalence to the general population in our area at the same time.

## Methods

### Study design and patients for Strasbourg National Reference Center for Autoimmune Diseases

We performed a cross-sectional phone survey, during which patients with autoimmune diseases followed at the National Reference Center for Rare Autoimmune Diseases of Strasbourg (CRMR RESO) were systematically contacted by phone by trained medical students, research assistants, and co-authors (up to 3 times in case they did not answer the initial call). In case of absence of response, the general practitioner and patient relatives were called in order to inquire about the patient health or whereabouts (move to another area/country, death and its cause). Patients with autoimmune diseases were identified from a comprehensive computerized list from the CRMR RESO. This list was extensively reviewed by the medical team and the clinical files checked when needed to ensure the inclusion of patients with a confirmed diagnosis of autoimmune disease (patients with suspected diseases were excluded). These diseases included systemic lupus erythematosus, Sjögren’s syndrome, systemic sclerosis, idiopathic inflammatory myopathies, juvenile idiopathic arthritis, vasculitis, overlap syndromes, primary antiphospholipid syndrome, sarcoidosis, Behçet’s disease, mixed connective tissue disorder, relapsing polychondritis, undifferentiated connective tissue disorder, Still’s disease, and Shulman’s disease. Inflammatory rheumatic diseases (e.g., rheumatoid arthritis and spondyloarthritis) were not included in our study. Following information regarding the methodology and general purpose of the study, patients who consented to participate were assessed during a phone survey using a standardized questionnaire which collected their demographic characteristics, known risk factors for COVID-19, potential exposure to SARS-CoV-2, preventive sick leave, symptoms of COVID-19, current treatments (including self- or medically prescribed changes in those treatments due to the pandemic), documented SARS-CoV-2 infection status (and the detailed means of confirmation), and main outcomes in case of SARS-CoV-2 infection. Reported flare of the autoimmune condition was evaluated by asking the patient if she/he experienced symptoms compatible with a flare and if so, which one. A reported flare was considered present if the reported symptoms were compatible with a flare of the disease. Patients were also sent information regarding the methodology, general purpose of the study, and a prescription for a SARS-CoV-2 serology to be conducted in their local laboratory. If they accepted to be serologically tested, they signed a written consent to participate to the study. The telephone survey took place from April 9, 2020, to July 2, 2020. Non-centralized serologic tests were conducted between May 18, 2020, and July 2, 2020. The study was approved by the ethics committee of Strasbourg (#CE-2020-50).

In order to compare the seroprevalence of SARS-CoV-2 infection in our cohort of patient with autoimmune disease to the seroprevalence in the general population of the Grand-Est area, we used the results of the seroprevalence study of Carrat et al. [3].

### Statistical analysis

For descriptive statistics, continuous variables were presented as median and interquartile range (IQR) and categorical variables as numbers and percentages. Comparison between groups was performed using the χ^2^ test (or Fisher’s exact test when appropriate) for categorical variables and the Mann-Whitney test for continuous variables. Multivariate analysis was performed to assess the association between documented COVID-19 cases (dependent variables) and potential predictors (p values < 0.10 in univariate analysis). All tests were bilateral, using an alpha risk of 0.05. Statistical analyses were performed using the software JMP 13 (SAS Institute, USA).

## Results

**Fig. 1:**
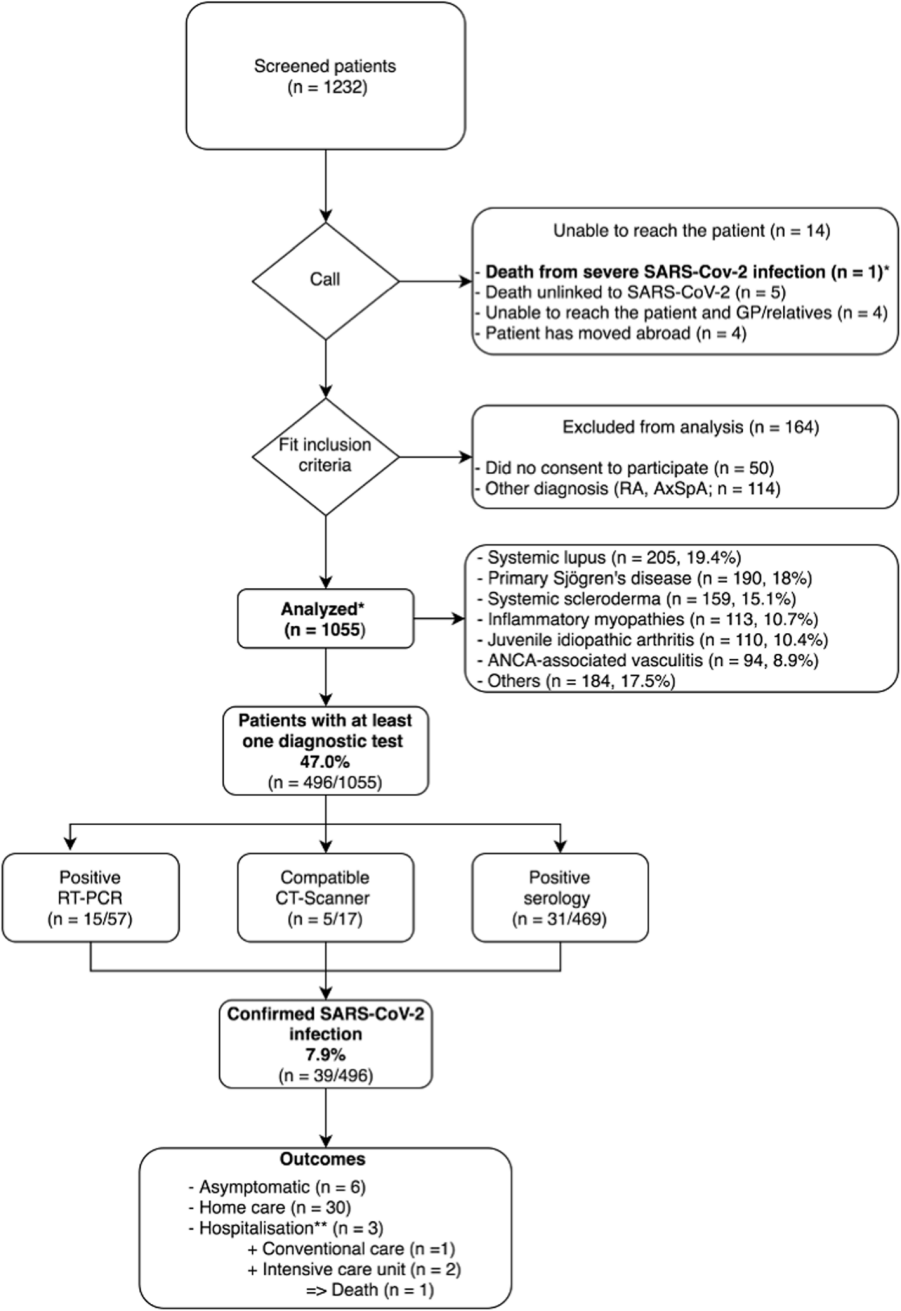
Flow chart of the study and prevalence and outcomes of confirmed SARS-CoV-2 infections. Single asterisk indicates that the unreached patient dead from SARS-CoV-2 was included in the outcome analysis. Double asterisks indicate that all hospitalized patients have been discharged home at the time of the study writing

Based on active cohorts followed at our National Reference Center, a total of 1232 patients were contacted by phone (Fig. 1). Fourteen patients could not be reached. Among them, general practitioner or relatives informed us of one death due to SARS-CoV-2 (included in the outcome analysis), 5 deaths of other causes, 4 moves abroad, and 4 patients for whom they could not provide information.

Fifty patients did not consent to participate; 114 patients were excluded because they did not have a confirmed autoimmune disease (flowchart Fig. 1).

In total, 1055 patients with a confirmed diagnosis of systemic autoimmune disease were included. There were 840 women (79.6%) with a median age of 53 years (IQR 37–65). Among them, 469 (44.5%) patients were tested for SARS-CoV-2 serology. Detailed demographic characteristics, autoimmune disease diagnosis, known risk factors for COVID-19, potential exposure to SARS-CoV-2, use of protective measures, symptoms of COVID-19, and current treatments are summarized in Table 1.

**Table 1:** Demographic characteristics, autoimmune disease diagnosis, known risk factors for COVID-19, potential exposure to SARS-CoV-2, use of protective measures, symptoms of COVID-19, current treatments and modifications, flares, and serological status for SARS-CoV-2

Patient characteristics	
**Age (years)**, median [IQR25-75]	53 (37–65)
**Female**, n (%)	840 (79.6%)
**AISD diagnosis, n (%)**	
*Lupus*	205 (19.4)
*Sjögren*	190 (18)
*Systemic sclerosis*	159 (15.1)
*IM*	113 (10.7)
*JIA*	110 (10.4)
*Vasculitis*	94 (8.9)
*Overlap syndrome*	84 (8.0)
*Primary APS*	33 (3.1)
*Sarcoidosis*	26 (2.5)
*Behçet*	15 (1.4)
*MCTD*	14 (1.3)
*Other*	12 (1.2)
**Benefited from preventive work cessation**, n (%)	96/852 (11.3)
**At least one contact with a confirmed COVID-19 case**, n (%)	63/1054 (6.0)
**Self-suspected SARS-CoV-2 infection (according to the patient him/herself)**, n (%)	201/1051 (19.1)
**Self-reported SARS-CoV-2 infection symptoms**, n (%)	/1054
*Fever*	89 (8.4)
*Chills*	62 (5.9)
*Myalgia*	86 (8.2)
*Cough/dyspnea*	133 (12.6)
*Thoracic pain/oppression*	39 (3.9)
*Gastroenteritis*	54 (5.1)
*Anosmia*	42 (4.0)
*Thrombosis*	1 (0.09)
**Risk factors for SARS-CoV-2 infection** (history of), n (%)	/1054
*Cancer*	49 (4.6)
*Cardiac failure*	34 (3.2)
*Myocardial infarction*	29 (2.7)
*Diabetes*	71 (6.7)
*Hypertension*	250 (23.7)
*Cerebrovascular event*	47 (4.5)
*Respiratory failure*	126 (12.0)
*Renal failure*	65 (6.2)
*Hepatic insufficiency*	43 (4.1)
**BMI (kg/m**^**2**^**)**, median [IQR25-75]	24.4 (21.2–28.7)
*BMI > 35*	53 (5.7)
*BMI > 40*	19 (2.1)
**Smoking status**, n (%)	/973
*Current smoker*	129 (13.3)
*Former smoker*	273 (28.1)
*Never smoker*	571 (58.7)
**Treatments**, n (%)	/1054
*NSAIDs*	81 (7.7)
*Glucocorticoids*	289 (27.4)
*Antimalarials (HCQ/CQ)*	262 (24.9)
*Immunosuppressive agents/biologics*	447 (42.4)
**Treatment modification due to pandemic**, n (%)	73/1054 (6.9)
**Reporting a flare-up of AISD** (self-diagnosed), n (%)	166/1054 (15.7)
**Serological status for SARS-CoV-2**, n (%)	
*Positive IgG and/or IgM*	31/469 (6.6)

*Other: relapsing polychondritis (n = 6), undifferentiation connective tissue disease (n = 3), Still’s disease (n = 2), Shulman’s disease (n = 1). Numbers between brackets: data availability

*Abbreviations*: AISD, autoimmune systemic disease; APS, anti-phospholipid syndrome; BMI, body mass index; JIA, juvenile idiopathic arthritis; IIM, idiopathic inflammatory-myositis; MCTD, mixed-connective tissue disorder; NSAIDS, non-steroidal anti-inflammatory drugs

Two hundred and one (19.1%) patients reported a self-suspected SARS-CoV2 infection. However, only 39 (7.9%) had proven SARS-CoV-2 infection (either through chest CT-scan [n = 5], nasopharyngeal RT-PCR [n = 15], or serology [n = 31]) among the 495 who underwent at least one of those 3 diagnosis modalities (Fig. 1). Among confirmed cases, 33 had clinical manifestations of COVID-19 [4] (6 asymptomatic patients were diagnosed through systematic serology testing). Of the 39 proven cases, 31 were managed by home care, 3 were hospitalized due to a need for oxygenation, 2 required admission to an intensive care unit, and one died. Among the 5 patients with SARS-Cov2 infection proven by CT-scan, all also had a positive RT-PCR and/or a positive serology.

Considering the 136 patients who reported having clinical signs suggestive of SARS-CoV-2 infection and who were serologically tested, 25 (18.4%) had a positive SARS-CoV-2 serology.

Overall, the seroprevalence of SARS-CoV-2 was 6.6% (31/469) which was numerically but not significantly lower to the Grand-Est general population estimated to be 9.0% (270/3434, p = 0.34) by Carrat et al. [3].

The frequency of SARS-CoV-2 infection (confirmed by chest CT-scanner, RT-PCR and/or serology) did not differ significantly between patients with preventive sick leave (3/52, 5.8%) and those without (30/397, 7.6%), p = 0.64.

Comparisons of patients’ characteristics with and without confirmed SARS-CoV-2 infection are shown in Table 2. Prevalence of positive COVID-19 serology in each autoimmune systemic disease is provided in supplementary Table 1.

**Table 2:** Demographic characteristics, autoimmune disease diagnosis, risk factors for SARS-CoV-2 infection, and current treatments

	Patients with a confirmed SARS-CoV-2 infection, n = 39	Non-infected patients n = 457	p
**Age (years)**, median [IQR25-75]	56.9 (47.6–65.9)	56.9 (43.5–67.2)	0.80
**Female**, n (%)	29 (74.4%)	363 (79.4%)	0.42
**Preventive work cessation**, n (%)	3 (9.1%)	49 (11.8%)	0.64
**At least one contact with a confirmed COVID-19 case**, n (%)	16 (42.1%)	27 (5.9%)	< 0.0001
**Self-suspected SARS-CoV-2 infection (n = 201) (according to the patient him/herself)**, n (%)	32 (84.2%)	122 (26.8%)	< 0.0001
**Risk factors for SARS-CoV-2 infection** (history of), n (%)^[1054]^			
*Cancer*	2 (7.9%)	25 (5.5%)	0.53
*Cardiac failure*	1 (2.6%)	14 (3.1%)	0.88
*Myocardial infarction*	0	15 (3.3%)	0.26
*Diabetes*	1 (2.6%)	30 (6.6%)	0.34
*Hypertension*	9 (23.7%)	126 (27.6%)	0.61
*Cerebrovascular event*	3 (7.9%)	24 (5.3%)	0.49
*Respiratory failure*	5 (13.2%)	70 (15.3%)	0.72
*Renal failure*	2 (5.3%)	36 (7.9%)	0.56
*Hepatic insufficiency*	2 (5.3%)	20 (4.4%)	0.80
**BMI (kg/m**^**2**^**)**, median [IQR25-75]	24.2 (21.3–28.2)	24.8 (21.5–28.7)	0.36
*BMI > 35*	2 (5.4%)	31 (7.7%)	0.62
*BMI > 40*	0	13 (3.2%)	0.27
**Smoking status**, n (%)			0.13
*Current smoker*	1 (2.8%)	65 (15.0%)	
*Former smoker*	13 (36.1%)	141 (32.5%)	
*Never smoker*	22 (61.1%)	228 (52.5%)	
***Smoking ever***	14 (38.9%)	206 (47.5%)	0.32
**Treatments**, n (%)^[1054]^			
*NSAIDs*	5 (13.2%)	28 (6.1%)	0.09
*Glucocorticoids*	9 (23.7%)	135 (29.6%)	0.44
*Antimalarials (HCQ/CQ)*	14 (36.8%)	131 (28.7%)	0.29
*Immunosuppressive agents/biologics*	15 (39.5%)	195 (42.6%)	0.70
**Treatment modification due to pandemic**, n (%)	10 (26.3%)	32 (7.0%)	< 0.0001
**Reporting a flare-up of AISD** (self-diagnosed), n (%)	13 (34.2%)	58 (12.7%)	0.0003
**Serological status for SARS-CoV-2**, n (%)			
*Positive IgG and/or IgM*	31/33 (93.9%)	0	< 0.0001

Patients in whom there was a change in the immunosuppressive treatment during the pandemic were more likely to report a flare of the autoimmune disease (48% [35/73] vs. 13.4% [131/981], p < 0.0001). Among patients with confirmed SARS-CoV-2 infection, flares were more frequent than in uninfected patients (26.3% [10/38] vs. 7.0% [32/457], p < 0.0001).

## Discussion

To date, there is no evidence that patients with rare autoimmune disease face more risk of contracting SARS-CoV-2 than individuals without such disease, nor that they have a worse prognosis [2, 5]. Our study involves a large single-center cohort of more than 1000 patients with rare autoimmune and systemic diseases in the time of SARS-CoV-2 pandemics. Its main strengths are a very little rate of missing clinical data (< 0.4%), its localization in one of the highest SARS-CoV-2 prevalence area in France, and the addition of serologic data. As summarized in Fig. 1, we focused on finding evidence for each patient in our cohort and understanding why we could not reach some of them. We were able to retrieve information (moves, deaths unrelated to COVID-19) and even one death because of a severe SARS-CoV-2 infection in a 78-year-old woman with primary Sjögren’s syndrome. Only 4 patients could not be reached even through their general practitioner and relatives. Our study has also limits. First, we cannot exclude that some of the 4 patients without information even through their general practitioner have had SARS-CoV-2 infection. However, they represent less than 0.4% of the cohort making it unlikely to significantly alter our conclusions. Second, this study was a telephonic survey and recorded information such as symptoms suggestive of SARS-Cov2 infection or disease flares were declarative and not confirmed through clinical examination. This limit could hardly be prevented since the study was conducted during the pandemics peak and the general lockdown, but we chose robust arguments to define SARS-CoV-2 infection (nasopharyngeal swab RT-PCR and/or chest CT-scan and/or serology) to avoid this caveat. Serology was performed between 1 and 4 months after the end of the 1st wave in Grand Est, a delay allowing the development of a humoral response against SARS-CoV-2 [6]. To note, all patients diagnosed using chest CT-scan were also positive for RT-PCR or serology, as chest CT-scan might have limited specificity in patients with systemic autoimmune disease [7]. Finally, only 27.9% (56/201) of symptomatic patients were tested with a nasopharyngeal swab RT-PCR. In fact, at the time of the study, testing capacities in France were very low, and RT-PCR was prioritized for severe patients and healthcare practitioners.

Completing other published studies [8], our results allow to estimate the prevalence of SARS-CoV-2 infection in a large group of patients with rare autoimmune and systemic diseases, 7.9% (39/496). Among them, 33 patients developed clinical manifestations, three patients were hospitalized (3/1054, 0.28%) including 2 in an intensive care unit (2/1055, 0.19%), and one died. These results appear comparable to the general population of the same area on July 2: 0.32% for hospitalization (17893/5.5 million) [9].

The numerically lower seroprevalence of SARS-CoV-2 (6.6% versus 9%) observed among patients with systemic autoimmune diseases compared to the local general population [3] may be explained by the fact that these patients rigorously followed the preventive and control measures or that some patients did not develop humoral immunity. In fact, two patients in our cohort with RT-PCR-confirmed SARS-CoV-2 infection had a negative serology more than 4 weeks after infection. One had been treated with rituximab/chloraminophen for a MALT lymphoma 4 years before, and one was currently treated with methotrexate/hydroxychloroquine for a rhupus diagnosis. While bearing in mind that our study is retrospective and declarative, our results do not support the prescription of preventive sick leave and lowering of immunosuppressive treatments, as underlined in many recommendations from scientific societies [2]. In France, people with significant immunosuppression/serious medical conditions were not asked to take additional precautions to protect themselves from COVID-19. All fragile people, at risk of severe COVID-19, were required to strictly adhere to the “barrier measures” and could be put on preventive sick leave if this was not possible at their workplace. It is possible that at an individual level, some patient did shield which would significantly affect their exposure to SARS-COV2. However, it is also the case for patients without immunosuppression (e.g., suffering from hypertension, cardiovascular diseases, and even osteoarthritis or osteoporosis) which also “shielded” on their initiative, therefore limiting the potential bias.

Due to the low number of confirmed cases, we could not analyze risk factors for infection or the impact of disease-modifying therapies on the risk of SARS-CoV-2-infection. Available data suggest that patients treated with traditional immunosuppressive drugs, bDMARDs or tsDMARDs, are not at increased risk of severe COVID-19 [10, 11] and that hydroxychloroquine has no significant protective effect [12].

Immunosuppressive treatment modification, whether due to proven infection or not, were significantly associated with self-declared disease flares but also with confirmed SARS-CoV-2 infections. Giving our results, it is impossible to know “which came first: the chicken or the egg?”. What we observed is that most patients modifying their immunosuppressive treatment did not have a confirmed SARS-CoV-2 infection (76.1%, 32/42). In addition, among patients with a confirmed SARS-CoV-2 infection, only about a quarter have modified their immunosuppressive treatment (26.3%, 10/38). Finally, of the 13 patients with a confirmed infection and who also experimented flares, 7 (53.9%) have modified their immunosuppressive treatments.

### Limitations

Since we did not ask this specific question about the reason for the treatment modification due to a possible shortage, we cannot exclude that some drug shortages may have impacted flare rates in our study. Finally, this study was led by the French national referral center for rare systemic diseases (CRMR RESO) which aims to study rare systemic diseases. Hence, more frequent diseases such as rheumatoid arthritis and spondyloarthritis were not included in our study.

Only 25 of 136 (18.4%) serologically tested patients who reported having clinical signs suggestive of SARS-CoV-2 infection had a positive serology. These data suggest that most patients reporting symptoms did not encounter the virus (or did not develop a humoral response for unexplained reasons). Patients’ perception of their health in time of pandemics may be greatly influenced by the media. A nocebo effect caused by the context of the pandemics may be responsible for non-specific symptoms (headache, dyspnea, cough, etc.) [7]. Moreover (and non-exclusively), a cognitive bias known as incorrect causal attribution bias makes one likely, in time of great stress induced by the pandemics, to attribute non-specific symptoms to a specific diagnosis such as COVID-19 infection, even without objective proof of it [13].

## Conclusion

This systematic survey of more than 1000 patients with rare systemic autoimmune diseases reports a low prevalence of proven SARS-CoV-2 infection of 7.9% and very rare severe infections that may be related to good compliance with prophylactic measures in these patients. Our study may help better tailor patient recommendation in the settings of the SARS-CoV-2 pandemics.

## Supplementary Information

**Additional file 1.** Supplementary table 1: comparison of the prevalence of a positive SARS-CoV-2 serology between rare autoimmune diseases.

## Data Availability

All data relevant to the study are included in the article or uploaded as supplementary information.
