# Supplementary material for: Incidence and predictors of COVID-19 and flares in patients with rare autoimmune diseases: a systematic survey and serological study at a national reference center in France
Source: Arthritis Res Ther. 2021 Jul 13;23:188. doi: 10.1186/s13075-021-02565-0 (PMC8276223; doi:10.1186/s13075-021-02565-0)
Supplement: Supplementary file 1 — Additional file 1. Supplementary table 1: comparison of the prevalence of a positive SARS-CoV-2 serology between rare autoimmune diseases. [file 13075_2021_2565_MOESM1_ESM.docx]

**Supplementary table 1: comparison of the prevalence of a positive SARS-CoV-2 serology between rare autoimmune diseases.**

| Disease | Positive serology (n/N) | % of positive serology, [CI95%] |
| --- | --- | --- |
| Behçet's | 0/7 | 0 [N/A] |
| IIM | 2/26 | 7.7 [1.0%-25.1%] |
| JIA | 0/2 | 0 [N/A] |
| Lupus | 7/111 | 6.3 [2.6-12.6] |
| MCTD | 0/5 | 0 [N/A] |
| Overlap syndrome | 1/41 | 2.4 [0.1-12.9] |
| Primary APS | 0/16 | 0 [N/A] |
| Relapsing polychondritis | 1/2 | 50 [1.3-98.7] |
| Sarcoidosis | 2/13 | 15.4 [1.9-45.4] |
| Shulmann’s | 0/1 | 0 [N/A] |
| Sjögren’s | 11/113 | 9.7 [5.0-16.8] |
| Still’s | 0/1 | 0 [N/A] |
| Systemic sclerosis | 4/72 | 5,6 [1.5-13.6] |
| UCTD | 1/2 | 50 [1.3-98.7] |
| Vasculitis | 2/57 | 3.5 [0.4-12.1] |
| Total | 31 | 6.6 [4.5-9.2] |

IIM, idiopathic inflammatory myositis; JIA, juvenile idiopathic arthritis; primary APS, primary anti-phospholipid syndrome; MCTD, mixed-connective tissue disease; UCTD, undifferentiated connective tissue disease. Exact confidence intervals were computed using the binomial law. N/A, non assessable.
